# Recommended Reference Genes for Quantitative PCR Analysis in Soybean Have Variable Stabilities during Diverse Biotic Stresses

**DOI:** 10.1371/journal.pone.0134890

**Published:** 2015-08-05

**Authors:** Raman Bansal, Priyanka Mittapelly, Bryan J. Cassone, Praveen Mamidala, Margaret G. Redinbaugh, Andy Michel

**Affiliations:** 1 Department of Entomology, Ohio Agricultural Research and Development Center, The Ohio State University, Wooster, OH, 44691, United States of America; 2 Center for Applied Plant Sciences, Ohio Agricultural Research and Development Center, The Ohio State University, Wooster, OH, 44691, United States of America; 3 Department of Biotechnology, University College of Science, Telangana University, Dichpally, Nizamabad, Telangana, 503322, India; 4 USDA-ARS Corn and Soybean Research Unit, and Department of Plant Pathology, Ohio Agricultural Research and Development Center, The Ohio State University, Wooster, OH, 44691, United States of America; ISA, PORTUGAL

## Abstract

For real-time reverse transcription-PCR (qRT-PCR) in soybean, reference genes in different tissues, developmental stages, various cultivars, and under stress conditions have been suggested but their usefulness for research on soybean under various biotic stresses occurring in North-Central U.S. is not known. Here, we investigated the expression stabilities of ten previously recommended reference genes (*ABCT*, *CYP*, *EF1A*, *FBOX*, *GPDH*, *RPL30*, *TUA4*, *TUB4*, *TUA5*, and *UNK2*) in soybean under biotic stress from *Bean pod mottle virus* (BPMV), powdery mildew (PMD), soybean aphid (SBA), and two‐spotted spider mite (TSSM). BPMV, PMD, SBA, and TSSM are amongst the most common pest problems on soybean in North-Central U.S. and other regions. Reference gene stability was determined using three software algorithms (geNorm, NormFinder, BestKeeper) and a web-based tool (RefFinder). Reference genes showed variability in their expression as well as stability across various stressors and the best reference genes were stress-dependent. *ABCT *and *FBOX *were found to be the most stable in soybean under both BPMV and SBA stress but these genes had only minimal to moderate stability during PMD and TSSM stress. Expression of *TUA4* and *CYP* was found to be most stable during PMD stress; *TUB4* and *TUA4* were stable under TSSM stress. Under various biotic stresses on soybean analyzed, *GPDH* expression was found to be consistently unstable. For all biotic stressors on soybean, we obtained pairwise variation (V_2/3_) values less than 0.15 which suggested that combined use of the two most stable reference genes would be sufficient for normalization. Further, we demonstrated the utility of normalizing the qRT-PCR data for target genes using the most stable reference genes validated in current study. Following of the recommendations from our current study will enable an accurate and reliable normalization of qRT-PCR data in soybean under biotic stress.

## Introduction

Soybean is the world’s major agricultural crop with multiple uses including as human and animal food, biofuel, feedstock, industrial products, and cosmetics [1]. The United States (U.S) is one of the leading producers of soybean, accounting for 32% of the world’s total production (284 million MT) with crop value of $42 billion [2]. However, soybean yield in the North-Central region, which accounts for 80% of the U.S crop, is adversely affected by various biotic and abiotic stresses [3]. Chief among the biotic stresses are invertebrate herbivores such as the soybean aphid (SBA, *Aphis glycines*) and microbial pathogens including various fungi and oomycetes (e.g. *Microsphaera diffusa* causing powdery mildew (PDM); *Phytophthora sojae* causing root and stem rot), and viruses [e.g. *Soybean mosaic virus* (SMV), *Bean pod mottle virus* (BPMV)]. The use of pesticides, a quick solution for managing pests, has resulted in sudden increase in input costs for soybean production in the region [4].

Host plant resistance (HPR) has been actively explored to manage these pests as it is economical and environmentally safe [5]. HPR employs resistance (R) genes whose products can recognize the pest attack and directly or indirectly initiate the plant defenses [6]. Several soybean R genes responsible for resistance to various pests have been identified. For example *Rmd*, *Rag*, *Rps*, and *Rsv* genes provide resistance to *M*. *diffusa*, *A*. *glycines*, *P*. *sojae*, and *SMV*, respectively. However, the durability of this resistance is threatened as pests evolve virulent strains or biotypes, which can break R-gene-based resistance [1]. Consequently, to develop sustainable HPR, understanding of soybean-pest interactions at the molecular level has become a primary research focus.

Molecular investigations into plant-pest interactions have shown that the most plant defenses, including those mediated by R genes, are induced at the transcriptional level after pest attack, rather than being constitutive [6,7]. Therefore, much of the research related to plant-pest interactions is focused on elucidating the key genes being induced and identifying the sub-cellular pathways involved in biotic stress responses. Consequently, the ability to accurately profile the expression of a large number of genes while simultaneously sensitive enough to detect subtle expression changes assumes greater significance for research aimed at understanding molecular plant-pest interactions.

Emergence of the real-time reverse transcription-PCR (qRT-PCR) technique has transformed research involving gene expression measurement and molecular diagnostics [8]. Due to its high sensitivity and reproducibility in measuring mRNA levels, qRT-PCR is a powerful tool for gene expression analysis [9–11] and has replaced the conventional mRNA quantification practices such as northern blot, semi quantitative PCR, *in situ* hybridization, and nuclease protection assay because it is less labor-intensive and time-consuming (no post-PCR processing), as well as possesses a higher resolution and a wider dynamic range [11,12].

In order to correct for variation among treatments due to differences in quantity and quality of original samples, handling and preparation of reagents, qRT-PCR assays generally require an expression standard [13]. Therefore, identification of suitable housekeeping gene(s) (HKG) for normalization is a pre-requisite for accurate quantification of mRNA transcripts under a given set of conditions [14]. HKGs, also called as reference or internal control genes, are considered to have stable expression across various biotic and abiotic stresses, tissues, and developmental stages as they are involved in primary functions of a cell [15,16]. Due to their assumed stable expression, HKGs are commonly recognized as internal controls for normalization in mRNA quantification analysis [11]. However, gene expression measurement under given experimental conditions and several normalization studies using qRT-PCR have revealed alterations (up- or down-regulation) in HKG expression; thus supporting the hypothesis that there is no reference gene suitable to all biological systems [16,17]. From the review of literature on suitable reference genes having been reported so far in plants and other organisms, it appears that a condition-specific reference gene for a given species needs to be identified for accurate mRNA quantification.

Reference genes for qRT-PCR analysis in soybean have been reported [13,18–23]. The majority of these studies have evaluated candidate genes for stability in different tissues or at developmental stages or from various cultivars or under stress conditions. However, the utility of the reference genes recommended in these studies for research on soybean under various biotic stresses, especially those occurring in North-Central region of U.S., is not known. Therefore, in the work reported here, we evaluated the stabilities of previously recommended reference genes in soybean plants under biotic stress from BPMV, PMD, SBA, and two‐spotted spider mite (TSSM, *Tetranychus urticae*). Since 2000, BPMV has emerged as a destructive pathogen of soybean, resulting in significant yield losses [24,25]. Similarly, the PMD is a common fungal disease of soybean in northern U.S and other parts. The SBA, a native of Asia, has become as a economic pest in North-Central U.S. and has inflicted serious losses to soybean production [4]. Over the last ten years, significant damage has been reported from TSSM outbreaks occurring in extended hot and dry weather conditions [26]. During each of these biotic stresses, we assessed the stabilities of recommended reference genes in soybean. Based on our results, we made recommendations on suitable reference genes for expression studies in soybean under biotic stress.

## Materials and Methods

### Plant genotype

The soybean plant introduction PI243540 was used in all experiments. PI243540 is a cultivated accession from Japan belonging to maturity group IV. PI243540 possesses *Rag2* and *Rmd_PI243540* for resistance to SBA (biotype 1 and 2) [27] and PMD [28], respectively. For each treatment, six soybean seeds were planted in a 14-cm-deep by 15-cm-diameter plastic pot filled with potting soil (Scotts’ Miracle-Gro, Marysville, OH). Pots were thinned to three healthy seedlings and raised in a growth chamber at 24°C, 50–70% relative humidity, and a photoperiod of 16:8 h (Light:Dark) [29].

### Pest cultures and soybean inoculation/infestation

All pest inoculations/infestations were performed as outlined below at the first trifoliate (V1) stage. For the SBA, PMD, and TSSM treatments, samples were collected 12 h after inoculation/infestation. For BPMV, samples were collected 4 days after inoculation. Both the lateral and the middle leaflets of the first trifoliate were collected; leaflets collected from three plants were pooled to constitute one biological replicate of a treatment, and there were three biological replications for each treatment. Samples were collected in liquid nitrogen and were immediately frozen at -80°C until further processing.

SBA: SBAs were obtained from a laboratory colony that originated from insects collected from Urbana (IL, USA; 40°06’N, 88°12’W) in 2000 [30]. At Ohio Agricultural Research and Development Center (OARDC, Wooster, OH), a laboratory colony of these insects is maintained on susceptible soybean seedlings [SD01-76R (2)] in a rearing room at 23–25°C and 15:9 h (Light:Dark) photoperiod. In the experimental set-up, each soybean plant was infested with about 40 SBA individuals (of various ages) using a camel hair brush.

BPMV: An Ohio isolate of BPMV was maintained on the susceptible soybean cv. Sloan by serial leaf-rub inoculation [31]. To generate experimental plants, inoculum made by grinding infected leaf tissue into 10 mM KHPO4, pH 7 (1:4 w/v) was mechanically inoculated onto soybean plants as described [32]. Treatment Controls consisted of PI243540 of soybean seedlings similarly inoculated with buffer. Following the sample collection, plants were maintained in the growth chamber. At 10 days post-inoculation, the BPMV symptoms were inspected to confirm the successful inoculations.

TSSM: A laboratory colony of TSSM was established by transferring mites collected from on *New Guinea impatiens* and Gerbera plants in an OARDC greenhouse Wooster, OH to soybean (PI243540). The TSSM colony was maintained on soybean in a growth chamber at 24°C and a photoperiod of 14:10 h (Light:Dark). For treatments, individual soybean plants were infested with about 60 TSSM individuals using camel hair brush.

PMD: Each experimental soybean seedling was inoculated with a copious amount of *M*. *diffusa* spores by brushing with a PMD infected leaf (from Wyandot, a soybean line susceptible to PMD). Additionally, fresh Wyandot plants were also inoculated which served as positive control. At two weeks post-inoculation, the PMD symptoms were seen on the Wyandot plants, thus confirming the successful inoculations.

### RNA extraction, cDNA synthesis, and qRT-PCR analysis

Frozen leaf samples from each treatment were grounded in liquid nitrogen with pestle and mortar to a fine powder followed by their processing for total RNA extraction using TRI reagent (Molecular Research Center Inc, Cincinnati, OH, USA), as per the protocol provided by the manufacturer [16]. RNA samples were treated with TURBO DNase (Applied Biosystems/Ambion, Austin, TX, USA) to remove any DNA contamination. Using iScript cDNA synthesis kit (Bio-Rad Laboratories, Hercules, CA, USA), first strand cDNA was prepared with 1 μg RNA [16]. To confirm the absence of genomic DNA contamination in RNA samples, the minus RT control reaction lacking the reverse transcriptase enzyme was performed alongside.

Reference genes for this study were selected from previous studies where these were found to have stable expression in different tissues, developmental stages, and under stress conditions [13,18–23] (Table 1). Gene specific primers for each reference gene were designed using the ‘PrimerQuest’ tool of Integrated DNA Technologies, Inc. (Coralville, IA). Structures of reference genes evaluated in the current study including the primer binding positions are presented in S1 Fig. Initially, the minus RT and plus RT cDNA samples were subjected to reverse transcription PCR to test the primer specificity and the genomic DNA contaminations, respectively (S2 Fig). The qRT-PCR reactions were performed with iQ SYBR green super mix on a CFX-96 thermocycler system (Bio-Rad, Hercules, CA, USA). Each qRT-PCR reaction was performed with 1 μl (250 ng) of cDNA, 1 μl (100 μM) of each primer and 5 μl of iQ SYBR green super mix in 10 μl total volume. Each reaction was done in duplicate in 96-well optical-grade PCR plates, sealed with optical sealing tape (Bio-Rad Laboratories, Hercules, CA). PCR amplifications were done with the following cycling conditions: one cycle at 95°C (3 min), followed by 40 cycles of denaturation at 95°C (30 seconds), annealing and extension at 55°C for 45 sec [16]. Finally, melt curve analyses were done by slowly heating the PCR mixtures from 55 to 95°C (1°C per cycle of 10 s) with simultaneous measurements of the SYBR green signal intensities. Amplification efficiencies (E) and correlation coefficients (R^2^) for each primer pair were calculated as described in Bio-rad’s *Real-Time PCR Applications Guide* (catalog #170–9799) [16].

**Table 1:** Description of reference genes tested for qRT-PCR studies in soybean under biotic stress.

Gene symbol	Locus	Locus description	**Function**	**Reference**
*ABCT*	Glyma.12G020500	ATP-binding cassette transporter	Translocation of various substrates	[19,21]
*CYP*	Glyma.12G024700	Cyclophilin	Protein folding	[13]
*EF1A*	Glyma.19G052400	Elongation factor-1-alpha	Translational elongation	[22,23]
*FBOX*	Glyma.12G051100	F-box only protein	Putative Mg^2+^ and Co^2+^ transporter Cord	[19,21]
*GPDH*	Glyma.19G082300	Glyceraldehydes-3 phosphate dehydrogenase	Glucose metabolic process	[13]
*RPL30*	Glyma.13G318800	60s Ribosomal protein L30	Structural constituent of ribosome	[19]
*TUA4*	Glyma.20G136000	Tubulin alpha-4	Structural constituent of cytoskeleton	[22]
*TUB4*	Glyma.19G127700	Tubulin beta-4	Structural constituent of cytoskeleton	[18,22]
*TUA5*	Glyma.05G157300	Tubulin alpha-5	Structural constituent of cytoskeleton	[18,20,22,23]
*UNK2*	Glyma.06G038500	Hypothetical protein	Unknown	[18,20,22,23]

### Stability analysis of reference genes

Three software algorithms (geNorm [14], NormFinder [33], BestKeeper [34]) and a web-based tool (RefFinder, www.leonxie.com/referencegene.php) were used to determine the stability of reference genes. geNorm calculated the M value; the lower value for ‘M’ is an indicative of a more stable expression or low variation [14]. The M value is calculated by a geometric averaging of all the reference genes used in the study and mean pairwise variation of a reference gene from other reference genes. It is important to note that the candidate reference genes showing a higher M value (M>1.5) are not considered for normalization studies [16]. NormFinder also determines the expression stability, but by taking into account the intra- and inter group variations for candidate reference genes [16,33]. NormFinder provides the stability value for each gene, which is a direct measure of the estimated expression variation enabling evaluation of the systematic error introduced when using the gene for normalization [33]. The BestKeeper program determines the stability of a candidate reference gene on the basis of the standard deviation (SD) of the Ct (cycle threshold) values [34]. The most stable reference genes are identified based on having the lowest SD. The RefFinder is a comprehensive tool which ranks tested candidate reference genes by integrating the output of geNorm, NormFinder, BestKeeper, and the comparative Ct method [35]. RefFinder allocates an appropriate weight to each gene on the basis of its ranking in each program and then calculates the geometric mean of their weights to obtain an overall ranking.

Relative expression values of genes in biological samples, calculated by equation 2^(-ΔCt)^, were used as input data for both geNorm and NormFinder whereas the input data for both BestKeeper and RefFinder were raw Ct values. Data points for SBA, TSSM, and PMD-treated soybean were compared to uninfested/uninoculated soybean whereas for BPMV-inoculated soybean, these were compared to mock.

### Expression validation of target genes

The primer sequences for two target genes designed using the ‘PrimerQuest’ tool of Integrated DNA Technologies, Inc. (Coralville, IA) are as follows: Glyma.05g184500: sense-TGAGAGCCCATTTACCACATAC, antisense- TAGGGAGGTGTAAGAGCAGAA; Glyma.06g147100: sense- CCTCCTCCGAGTTCATGTTATC, antisense- CGATTCAGTTTCAGTGCTTTGT. The RNA extraction, cDNA synthesis, and qRT-PCR reaction were performed as described in the previous sections. The target gene expression data was normalized using the geometric mean values calculated for the reference gene pairs [14]. Relative expression level and fold change were determined using comparative Ct method 2^(-ΔCt)^ [36]. Statistical analysis was performed using t-test through the SigmaPlot 13.0 (Systat Software, Inc. San Jose, CA, USA).

## Results

### qRT-PCR assay optimization

Initially for each primer pair outlined in Table 1, we performed reverse transcriptase-PCR amplifications on pooled cDNA which yielded a specific fragment of expected size (S2 Fig). Further, the melting curve analysis (65°C to 95°C in increments of 0.5°C every 5 s) in the qRT-PCR reaction showed the presence of a single peak for each primer pair indicating an absence of nonspecific product amplification (S3 Fig). The amplification efficiencies for all primer pairs except *TUA5* ranged between 87.58% and 111.60% and the correlation coefficient (R^2^) for all primer pairs were greater than 0.9900 (Table 2). Taken together, these results indicate the qRT-PCR assays were highly specific and efficient.

**Table 2:** Primer sequences and amplicon characteristics of reference genes tested for qRT-PCR studies in soybean.

Gene symbol	Primer sequences	Amplicon length (bp)	Product melting temperature (°C)	Amplification efficiency E (%)	Correlation coefficient (R^2^)
*ABCT*	CTTTGCTTTTATTCCGAATGG	166	79.5	90.28	0.9988
	GCCTGCTTCAGATAAAATAGAT				
*CYP*	GTCGAGGGGATGGACGTC	158	85.0	105.13	0.9967
	CGACACATTCAGAGCCACCGA				
*EF1A*	CGTGGTTACTCCTTTATAGTAG	131	78.0	95.60	0.9972
	TGACCACCGCCGATCAAGAAC				
*FBOX*	GAAAGCAGAAAGATGGGGTTGG	99	80.0	103.69	0.9989
	CACACACGCCACTCTCGCAA				
*GPDH*	CGGTGCTGCTAGAGGATGG	138	83.0	99.34	0.9994
	CCCAGAAGCGCCAAGCACA				
*RPL30*	CAATGCTGCACTTAATTTTTGCCG	110	77.0	93.74	0.9995
	GAAGAACACATCATTCACATTAAT				
*TUA4*	TGTGTACCAGTACCGATGTGGT	150	79.5	111.60	0.9987
	GCACTCCCGTGCCCGTAA				
*TUB4*	TTCTCTGCACTCTTCATCAAGCTC	117	83.0	95.08	0.9988
	CACACCACTTCCCAGAACTTG				
*TUA5*	CGCTTCGCAAATCTCAATCGAATC	145	84.0	74.45	0.9987
	GAAGGCATCATGCCGTCGG				
*UNK2*	CTGATCACATTCACCGCCGA	109	79.0	87.58	0.9955
	AGTGACAAGCTTGCGCTACC				

### Wide ranging expression for reference genes

**Fig 1:**
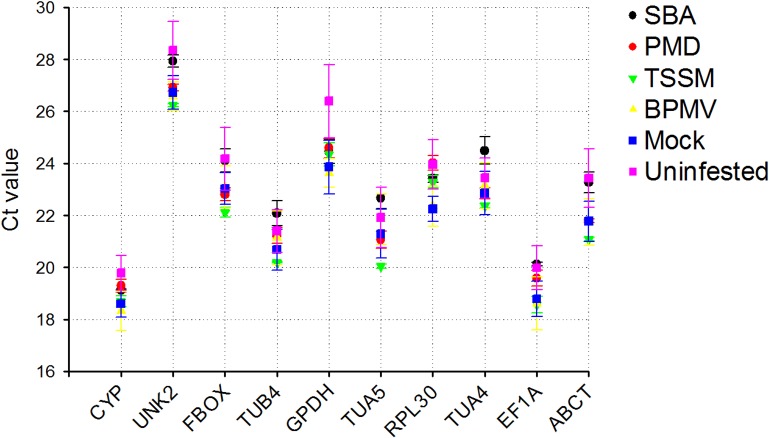
Ct values for reference genes in control and infested soybean plants. Details on tested reference genes are provided in Table 1. Each point represents the Mean± Standard Deviation of Ct values for three biological replications in each treatment. BPMV: *Bean pod mottle virus*; PMD: powdery mildew; SBA: soybean aphid; TSSM: two‐spotted spider mite.

The expression profiles of qRT-PCR products for all primer pairs in each experiment are shown in Fig 1. *CYP* and *EF1A* were highly expressed with mean Ct values ranging between 17 and 21 whereas *UNK2* was the least expressed gene with mean Ct value between 26 and 30. All tested genes showed expression variability across various samples as evident from a wide range of Ct values obtained. Genes such as *CYP* and *TUB4* showed relatively smaller variation (below 2 cycles) in their expression across various samples, while others like *GPDH* had the highest expression variation (nearly 5 cycles). Our selected genes had the most stable expression and had been recommended for use in qRT-PCR analysis in different soybean tissues, developmental stages, and under stress conditions [13,18–23]; however the variability and the wide range in their expression deemed it necessary to select a suitable reference to normalize soybean gene expression under biotic stress.

### geNorm analysis

**Fig 2:**
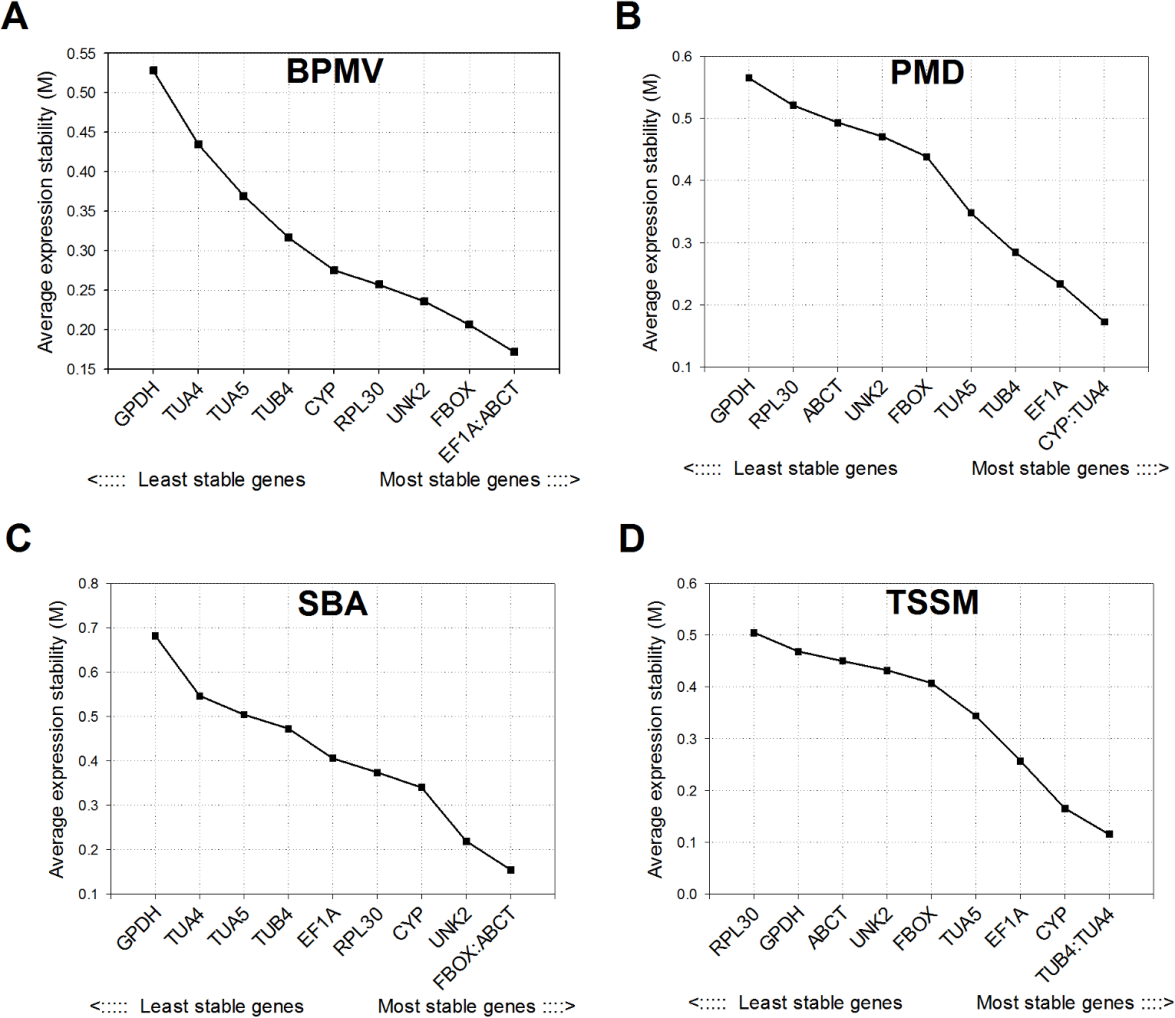
Average expression stability (M) and ranking of reference genes in soybean as calculated by geNorm. M values and rankings are indicated for soybean under stress by **A.** *Bean pod mottle virus* (BPMV), **B.** powdery mildew (PMD), **C.** soybean aphid (SBA), and **D.** two‐spotted spider mite (TSSM). Details on tested reference genes and primer sequences are provided in Tables 1 and 2, respectively.

geNorm algorithm calculated that following BPMV inoculation, the average expression stability values (M) for *ABCT* and *EF1A* were the lowest (0.1714) and that for *GPDH* was highest (0.5283), suggesting that *ABCT* and *EF1A* had the most stable expression and that *GPDH* had the least stable expression (Fig 2A). Nearly identical results were obtained following the SBA attack on soybean; as ABCT and FBox were the most stable (M value 0.1545). GPDH was the least stable during SBA attack as well (M value 0.6820) (Fig 2C). On the other hand, *ABCT* was among the least stable genes both in case of PMD (M value 0.4928) and TSSM (M value 0.4500) attack whereas *TUA4* was the most stable (PMD M value 0.1725 and TSSM M value 0.1154) (Fig 2B and 2D). Besides *TUA4*, *CYP* and *TUB4* were the most stable following the PMD and TSSM attack, respectively. *GPDH* (M value 0.5650) remained the least stable following the PMD attack whereas *RPL30* (M value 0.5042) was the least stable following the TSSM attack on soybean. The M value calculated for all the reference genes assayed in the current study was less than 1.5, thus suggesting the appropriateness of all genes for normalization consideration in soybean under biotic stress.

### NormFinder analysis

**Fig 3:**
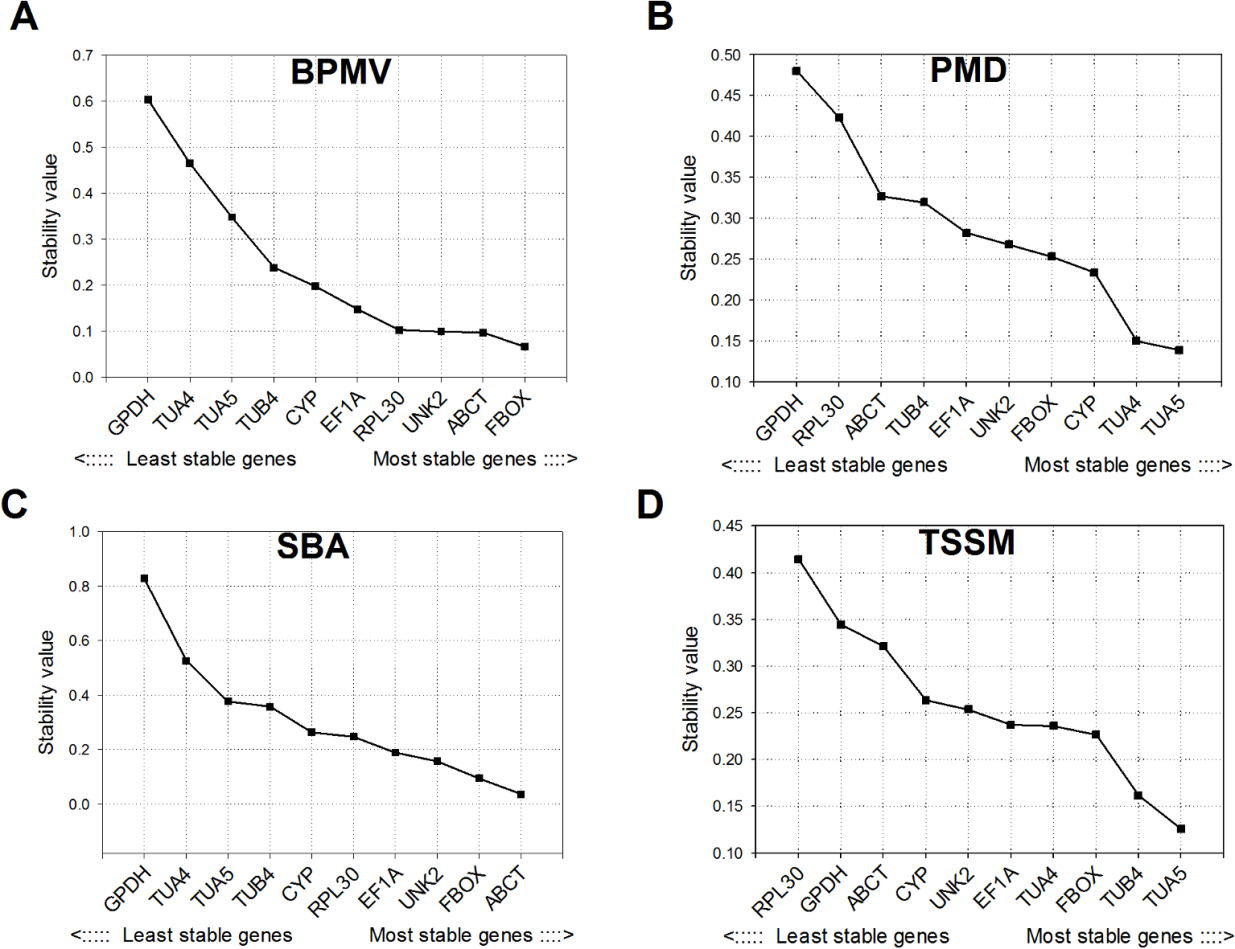
Stability values of reference genes in soybean calculated using NormFinder. Stability values are indicated for soybean under stress by **A.** *Bean pod mottle virus* (BPMV), **B.** powdery mildew (PMD), **C.** soybean aphid (SBA), and **D.** two‐spotted spider mite (TSSM). Details on tested reference genes and primer sequences are provided in Tables 1 and 2, respectively.

Following the BPMV and SBA attack, *ABCT* and *FBOX* were found to be most stable in soybean (Fig 3A and 3C). Both these genes have lowest stability value i.e. lowest variation of expression among all genes. In case of PMD and TSSM attack *TUA5* and *TUA4* were most stable as revealed by NormFinder (Fig 3B and 3D). Similar to geNorm analysis results, the *GDPH* was the least stable following the BPMV, PMD, and SBA attack whereas *RPL30* was the least stable following the TSSM attack (Figs 2 and 3).

### BestKeeper analysis

The BestKeeper analyses indicated that following the BPMV attack on soybean, *UNK2* and *RPL30* were the top ranked genes with highest stability, and *TUA5* was the bottom ranked gene with least stability (Table 3). In case of PMD attack on soybean, the most stable genes were *CYP* and *TUB4*. The *CYP* was also among the top ranked for SBA and TSSM attack on soybean. *EF1A* and *RPL30* were the top ranked genes with highest stability following the SBA and TSSM attack on soybean, respectively. Similar to geNorm and NormFinder analysis, *GPDH* was the least stable (having the highest SD) among all tested genes during PMD and SBA stress on soybean. Furthermore, the SD values of *GPDH* (during PMD, SBA, and TSSM stress), *FBOX*, *UNK2*, and *ABCT* (all during TSSM stress) surpassed the cutoff value of 1 which suggested that these genes are highly unstable across all treatments for qRT-PCR normalization.

**Table 3:** BestKeeper ranking of ten reference genes tested for qRT-PCR studies in soybean under biotic stress.

Rank	BPMV^a^		PMD^b^		SBA^c^		TSSM^d^	
	Gene	SD^e^	Gene	SD	Gene	SD	Gene	SD
1	*UNK2*	0.44	*CYP*	0.35	*EF1A*	0.37	*RPL30*	0.48
2	*RPL30*	0.47	*TUB4*	0.39	*CYP*	0.41	*CYP*	0.55
3	*FBOX*	0.51	*TUA4*	0.42	*RPL30*	0.46	*TUA4*	0.58
4	*CYP*	0.51	*EF1A*	0.43	*UNK2*	0.52	*TUB4*	0.62
5	*GPDH*	0.54	*RPL30*	0.49	*ABCT*	0.52	*EF1A*	0.71
6	*TUA4*	0.62	*TUA5*	0.58	*TUB4*	0.55	*TUA5*	0.93
7	*ABCT*	0.63	*FBOX*	0.83	*FBOX*	0.59	*FBOX*	1.06
8	*EF1A*	0.64	*UNK2*	0.86	*TUA4*	0.63	*UNK2*	1.09
9	*TUB4*	0.68	*ABCT*	0.89	*TUA5*	0.69	*GPDH*	1.12
10	*TUA5*	0.73	*GPDH*	1.03	*GPDH*	1.08	*ABCT*	1.18

Soybean biotic stress due to

^a^BPMV: *Bean pod mottle virus*

^b^PMD: powdery mildew

^c^SBA: soybean aphid

^d^TSSM: two‐spotted spider mite

^e^SD refers to the standard deviation.

### RefFinder analysis

The final ranking calculations based on the combined algorithm values found *FBOX* as the most stable soybean genes during the BPMV stress followed by *ABCT* and *UNK2* (Table 4). However, *TUA4* was the most stable during PMD stress followed by *CYP* and *TUA5*. For SBA stress, the order of the three best reference genes was as follows: *ABCT*, *FBOX*, and *EF1A* whereas for TSSM, it *was TUB4*, *TUA4*, and *TUA5*.

**Table 4:** RefFinder ranking of ten reference genes tested for qRT-PCR studies in soybean under biotic stress.

Rank	BPMV^a^		PMD^b^		SBA^c^		TSSM^d^	
	Gene	GM^e^	Gene	GM	Gene	GM	Gene	GM
1	*FBOX*	1.73	*TUA4*	1.57	*ABCT*	1.50	*TUB4*	1.68
2	*ABCT*	2.30	*CYP*	1.73	*FBOX*	2.30	*TUA4*	2.63
3	*UNK2*	2.45	*TUA5*	2.78	*EF1A*	3.13	*TUA5*	2.78
4	*RPL30*	3.56	*EF1A*	4.56	*UNK2*	3.22	*CYP*	4.14
5	*EF1A*	3.76	*TUB4*	4.60	*CYP*	4.12	*FBOX*	4.41
6	*CYP*	5.42	*FBOX*	5.09	*RPL30*	4.40	*EF1A*	4.95
7	*TUB4*	7.45	*UNK2*	6.12	*TUB4*	6.74	*RPL30*	5.62
8	*TUA4*	8.13	*RPL30*	7.77	*TUA5*	8.24	*UNK2*	6.40
9	*GPDH*	8.41	*ABCT*	7.97	*TUA4*	8.74	*ABCT*	8.46
10	*TUA5*	8.46	*GPDH*	10.00	*GPDH*	10.00	*GPDH*	9.00

Soybean biotic stress due to

^a^BPMV: *Bean pod mottle virus*

^b^PMD: powdery mildew

^c^SBA: soybean aphid

^d^TSSM: two‐spotted spider mite

^e^GM refers to the geometric mean.

### Optimal number of reference genes for normalization

**Fig 4:**
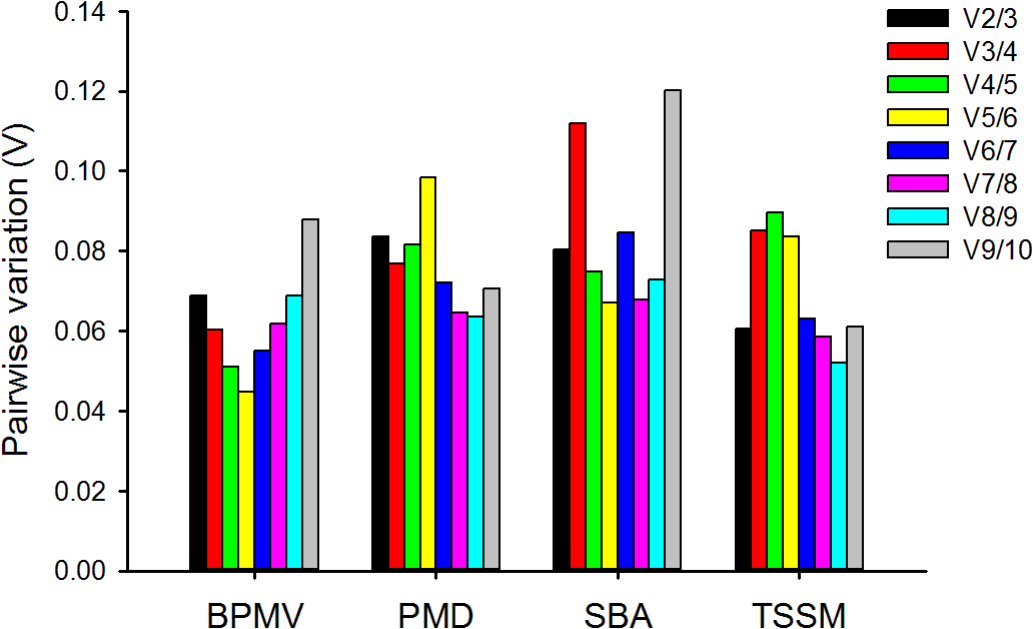
Pairwise variation (V) analysis of the reference genes in soybean using geNorm. The pairwise variation (V_n/n+1_) between the normalization factors NF_n_ and NF_n+1_ (shown along x-axis) is calculated to determine the optimal number of reference genes for normalization in soybean under stress by *Bean pod mottle virus* (BPMV), powdery mildew (PMD), soybean aphid (SBA), and two‐spotted spider mite (TSSM). Each bar indicates change in normalization when adding reference genes stepwise according to rankings in Fig 2.

Though a single reference gene which is stable and has moderate to high expression is sufficient for quantifying mRNA transcript levels, the use of more than one reference gene for effective normalization of gene expression data is suggested [14]. The optimal number of reference genes required for normalization under a given set of experimental conditions can be obtained from the pairwise variation (V). Vandesompele et al. [14] proposed a cutoff V value of 0.15, below which the inclusion of other reference genes are not required. Accordingly in all biotic stressors on soybean, the pairwise variation of V_2/3_ was less than 0.15 (Fig 4) which means that the addition of third reference gene will not further increase the statistical significance obtained for the first reference gene pair employed. Thus, the combined use of the two most stable reference genes would be enough for normalization.

### Usefulness of identified reference genes

**Fig 5:**
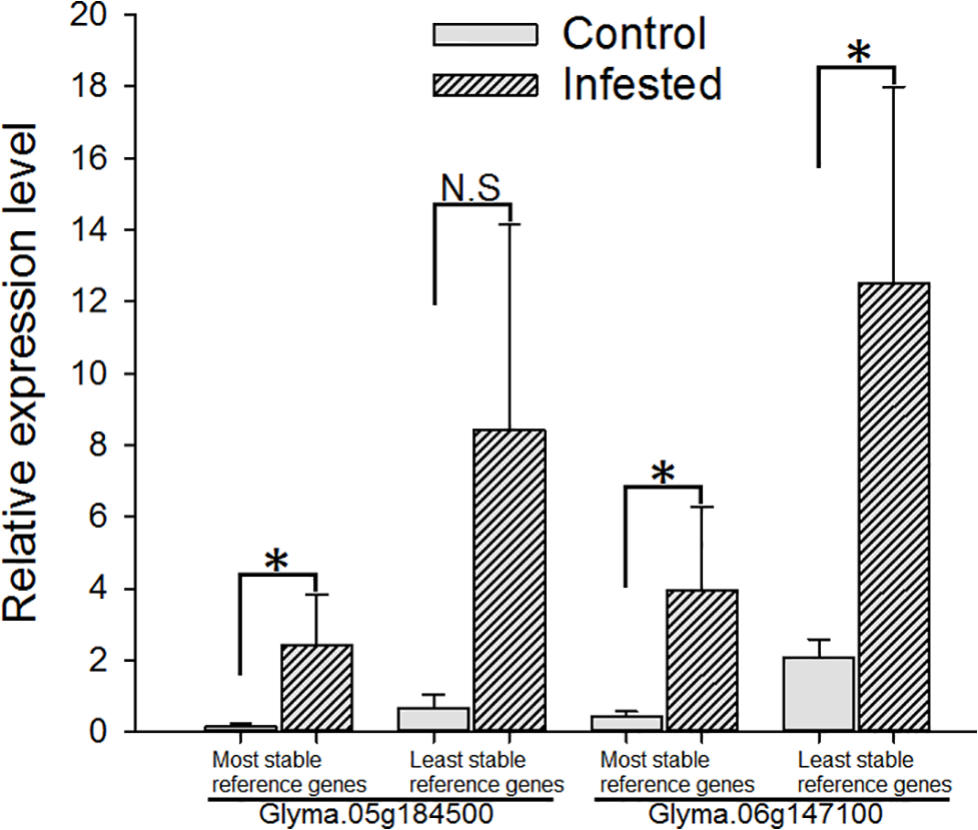
Usefulness of identified reference genes in detecting target gene expression. Relative expression levels (Mean± Standard Deviation) of two target genes (Glyma.05g184500 and Glyma.06g147100) in soybean under biotic stress by soybean aphid (SBA) are shown. Expression values of both target genes were normalized with respect to the most stable gene pair (*ABCT* and *FBOX*) and the least stable gene pair (*TUA4* and *GPDH*) during SBA stress in soybean (see Table 4). The asterisk (*) represents a significant difference (*P* < 0.05) in SBA-infested soybean compared to uninfested control. N.S: No significant difference (*P* > 0.05).

To demonstrate the utility of identified stable reference genes in validating the gene expression results obtained by high-throughput sequencing, we measured the expression of target genes in soybean under biotic stress. From soybean RNA-Seq data, two target genes, both encoding for WRKY transcription factors were selected. In general, the WRKY transcription factor genes are best known for their role in regulating the plant’s defense response to biotic stresses [37]. Our RNA-Seq experiment showed that following the SBA infestation, both the selected genes have enhanced expression compared to control plants (R. Bansal, unpublished data). For the purpose of comparison, expression values of target genes were normalized with respect to the most stable gene pair (*ABCT* and *FBOX*) and the least stable gene pair (*TUA4* and *GPDH*) during SBA stress in soybean. In case of normalization with the most stable gene pair, expression pattern of both target genes concurred with that observed in RNA-Seq analysis (Fig 5). However, in case of normalization with the least stable gene pair, one target gene (Glyma.06g147100) showed enhanced expression during SBA-stress, thus consistent with RNA-Seq results; but the other target gene (Glyma.05g184500) was shown to be unaffected during SBA-stress; thus contradicting with results obtained through the RNA-Seq and through the normalization with most stable gene pair.

## Discussion

Our long-term research goal is to decipher molecular interactions between soybean and its pests so as to identify the gene(s) and/or factors responsible for resistance. To precisely decipher the mechanisms driving biotic stress responses and to determine the key stress response genes, stably expressed reference genes are required for qRT-PCR analysis, which has many applications including high-throughput expression data (e.g. RNA-Seq) validation. Our results indicate that a subset of reference genes previously identified for different soybean tissues, developmental stages, cultivars, and stress conditions [13,18–23], can be used as reference genes to investigate the responses of soybean to biotic stresses occurring in North-Central region of U.S. We have demonstrated the utility of normalizing the qRT-PCR data for target genes using the most stable reference genes which were validated in this study. Our results suggested that qRT-PCR data normalization using the least stable reference genes may or may not be accurate (Fig 5).

Our results suggest that no single reference gene is best for expression studies on soybean under all biotic stresses. However, the rankings of some reference genes were similar between some biotic stresses. For example, *ABCT* and *FBOX* were most stably expressed in soybean during both the responses to BPMV and SBA as revealed by geNorm (Fig 2), NormFinder (Fig 3) and comprehensive RefFinder tool (Table 4). Previously, both *ABCT* and *FBOX* have been identified as the most stably expressed genes in soybean tissues, developmental stages, and under salt, dehydration, cold, abscisic acid, hormone and wounding stress [18,19,21]. Further, *ABCT* and *FBOX* were the most stably expressed during infection by Asian soybean rust (*Phakopsora pachyrhizi*) and nodulating bacteria (*Bradyrhizobium japonicum*) [21]. However in our study, these genes had the least to moderate stability during PMD and TSSM stress. Besides, *UNK2* was ranked the third most stable gene during the BPMV stress in soybean (Table 4) which is consistent with its performance during the stress by another virus i.e. *soybean mosaic virus* [22].

Based on comprehensive analysis, *TUA4* possessed highest stability during PMD stress and was the second most stable during TSSM stress. *CYP* was also highly stable during PMD stress. Earlier, *CYP* was reported to the most stable in soybean during stress by velvetbean caterpillar (*Anticarsia gemmatalis*) as well as in various tissues, developmental stages, cultivars, and in leaves on different stem nodes [13,23]. *TUB4* was the most stable during TSSM stress while this gene was also found to be stable during drought stress [22]. *TUB4* stability during both the drought and TSSM stresses is interesting as TSSM outbreak on soybean is more likely to occur during drought. Interestingly, *TUA5* appears to be the gene which remains unaffected during some but not all biotic stresses in soybean. In current study, it was ranked the third most stable gene during both the PMD and TSSM stresses (Table 4) and previously, this gene has shown to be the most stable during biotic stress by root-knot nematode (*Meloidogyne incognita*) [23].

Our results suggest no consensus on reference genes’ stability rankings among different software algorithms. However across all biotic stressors on soybean, NormFinder and geNorm analyses gave nearly identical results (Fig 2 and Fig 3), but BestKeeper yielded highly dissimilar rankings of reference genes for each of the biotic stressors (Table 3). The disparate ranking of candidate reference genes using different software algorithms has been frequently observed in several reference gene validation studies reported earlier [16].

Mining the large scale transcriptomic datasets (e.g. RNA-Seq, microarray) for reference genes and subsequent qRT-PCR validation provide superior set of reference genes for functional studies in any plant species [38]. In one such study on soybean, Libault et al [21] identified more than 200 putative reference genes through microarray experiments. Further evaluation of 18 of these putative reference genes revealed four genes which were deemed to be the most stable in diverse experimental conditions. These four genes included *ABCT* and *FBOX* which were also found to be the most stable during BPMV and SBA stresses in soybean in the current study (Table 4). Overall, most of the reference gene mining studies have largely been conducted in a few model plants (e.g. arabidopsis, rice, tomato) where several extensive transcriptomic datasets are available for a particular tissue, developmental stage, treatment, environmental condition etc. [38–41]. Recently, genome-wide transcriptome studies in soybean have been performed but these datasets have rarely been explored for identification of stable reference genes [42–44]. One possible reason for non-exploration of reference gene studies based on soybean transcriptome is the lack of accessibility to the complete gene expression datasets since data only on target response genes are made available [44].

## Conclusions

In the present study, we determined the expression stabilities of ten previously recommended reference genes in soybean under biotic stress by BPMV, PMD, SBA, and TSSM which are amongst the common pest problems on soybean in North-Central U.S. and other regions. Reference genes showed variability in their expression as well as stability across various stressors and the best reference genes were stress-dependent.

Taken together, using different software algorithms and considering comprehensive analysis results, we recommend that the following gene pairs are the best for use as reference genes under specific stress in soybean:

1. The *ABCT* and *FBOX* pair is the best for measuring gene expression differences between mock/control and infested plants under BPMV and SBA stress conditions.
2. For studying expression changes by PMD stress, *TUA4* and *CYP* is the best reference gene pair.
3. For TSSM stress, *TUB4* and *TUA4* should be used as reference genes for comparison between infested and control soybean plants.

In the case of different soybean stressors, the identified reference genes in the current study may possibly serve as ideal candidates for testing in that particular condition.

## Supporting Information

S1 Fig Structures of tested reference genes and corresponding primer binding locations. (BMP)

S2 Fig Reverse transcription PCR to test primer specificity and genomic DNA contaminations. Results of RT-PCR (35 amplification cycles) on plus RT cDNA (top) and minus RT cDNA (bottom) samples are presented for primer pairs used to amplify tested reference genes in soybean under biotic stress. More details on reference genes, primers, and amplicons are provided in Tables 1 and 2.
(BMP)

S3 Fig Melting curve analyses to test primer specificity. The melting curves are presented for primer pairs used to amplify tested reference genes in soybean under biotic stress. Details on tested reference genes and primer sequences are provided in Tables 1 and 2, respectively.
(PDF)
