# Supplementary figures and images for: Recommended Reference Genes for Quantitative PCR Analysis in Soybean Have Variable Stabilities during Diverse Biotic Stresses
Source: PLoS One. 2015 Aug 5;10(8):e0134890. doi: 10.1371/journal.pone.0134890 (PMC4526470; doi:10.1371/journal.pone.0134890)

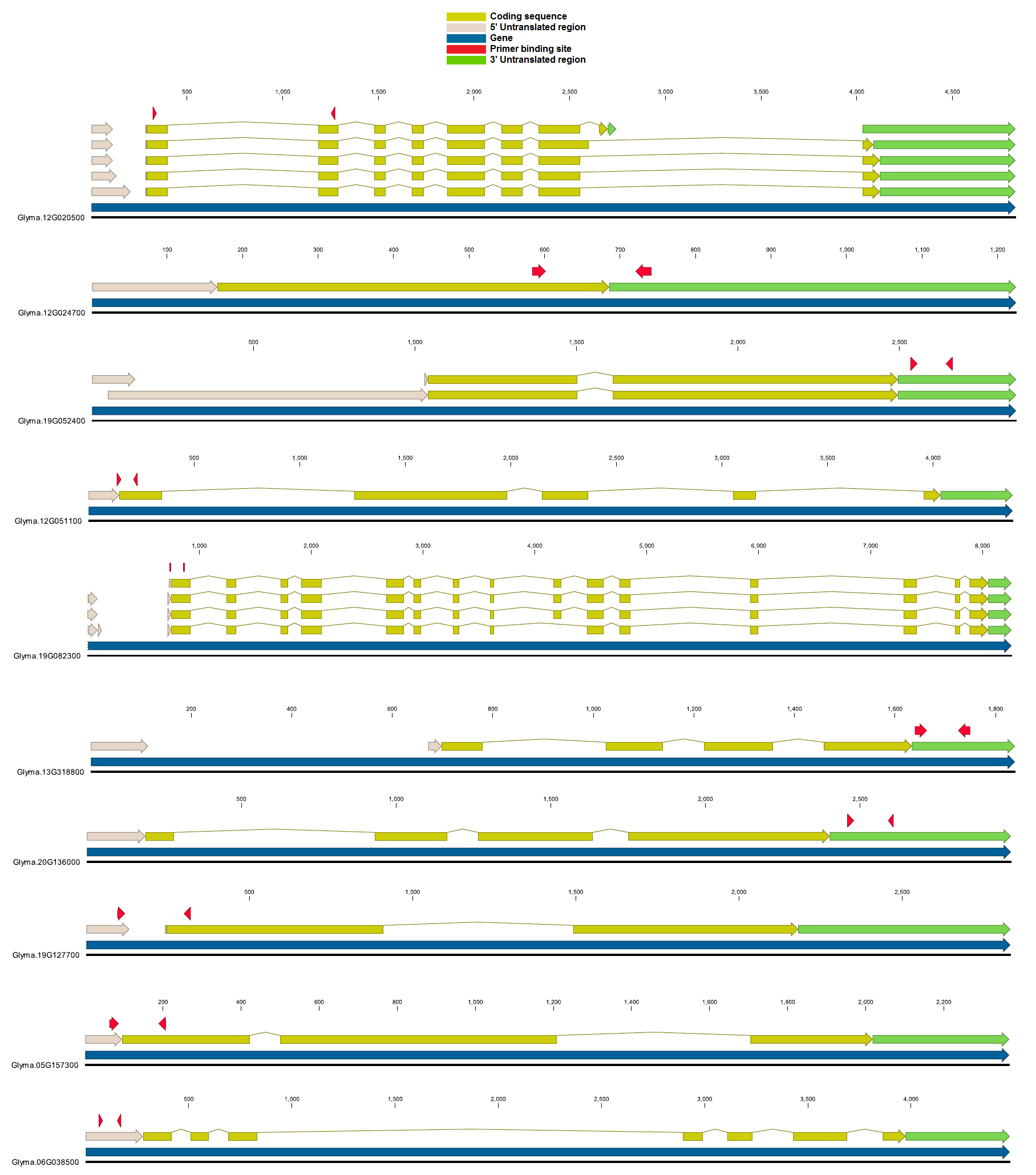

Supplement: S1 Fig — (BMP) [file pone.0134890.s001.bmp]

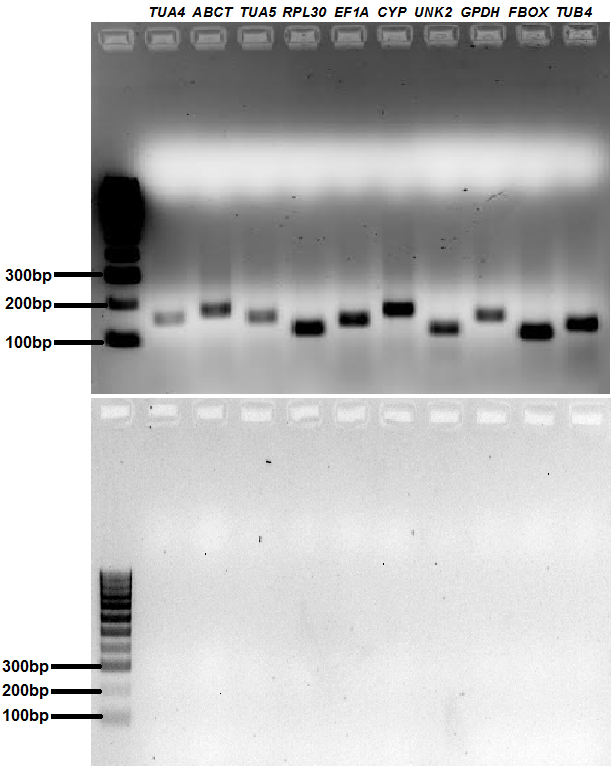

Supplement: S2 Fig — Results of RT-PCR (35 amplification cycles) on plus RT cDNA (top) and minus RT cDNA (bottom) samples are presented for primer pairs used to amplify tested reference genes in soybean under biotic stress. More details on reference genes, primers, and amplicons are provided in Tables 1 and 2. (BMP) [file pone.0134890.s002.bmp]

## CYP

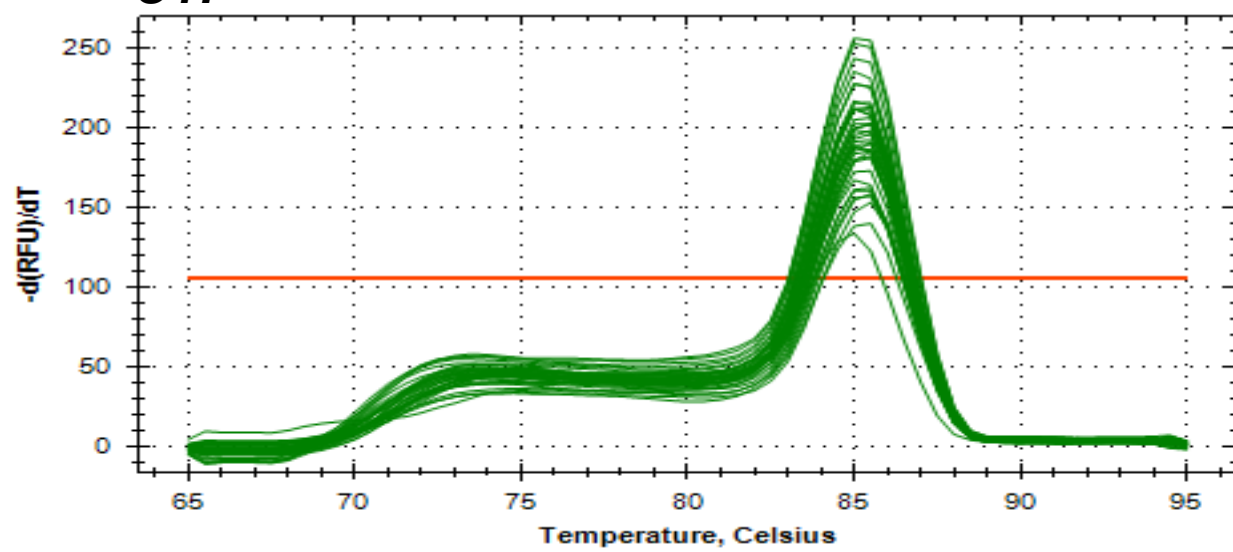

## UNK2

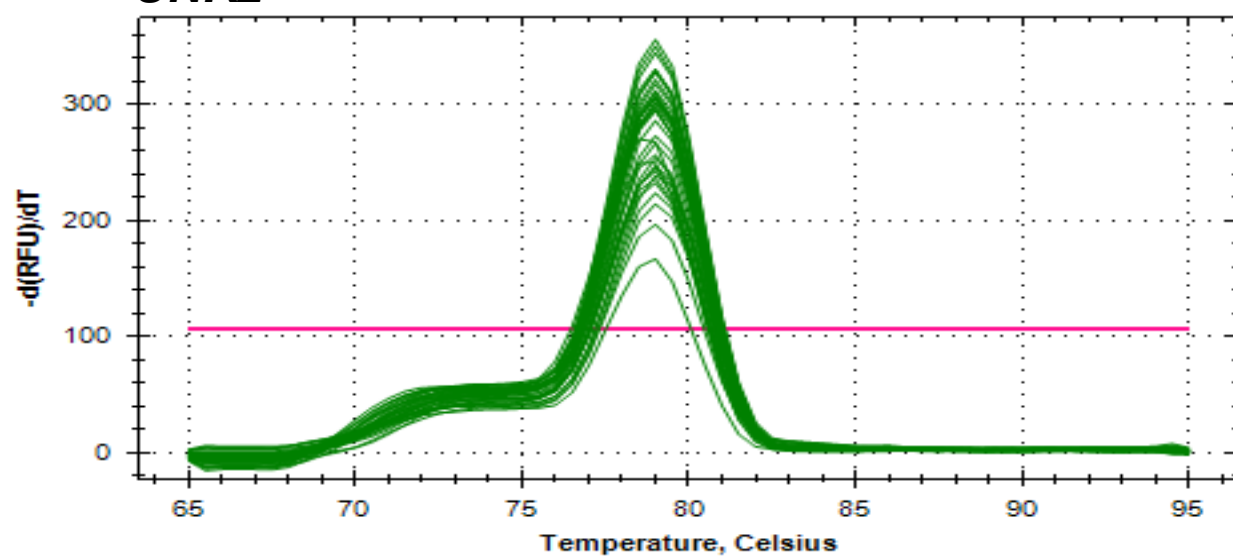

### ***FBOX***

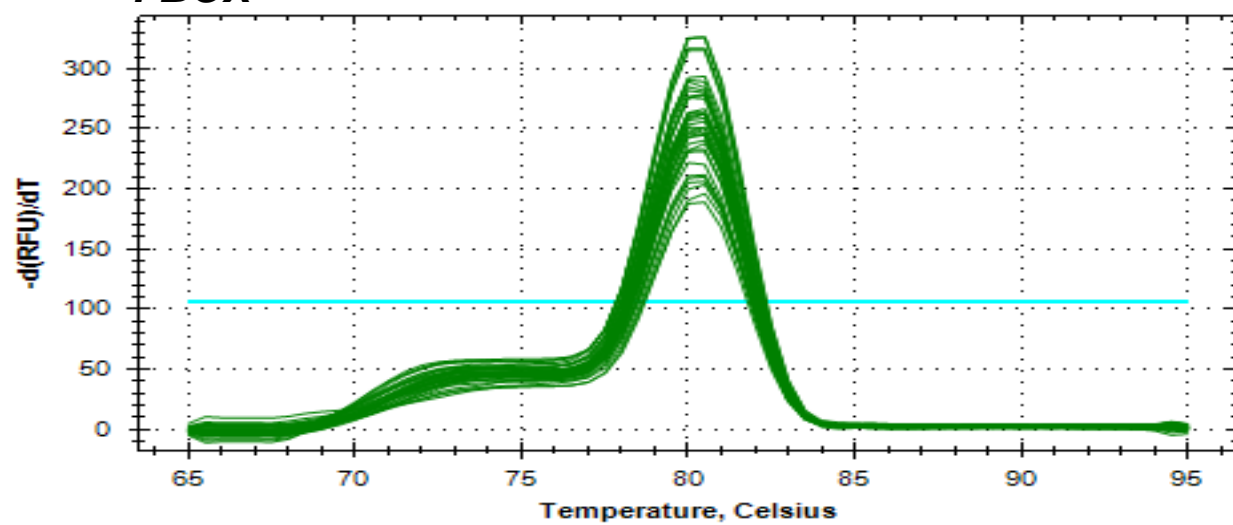

### ***TUB4***

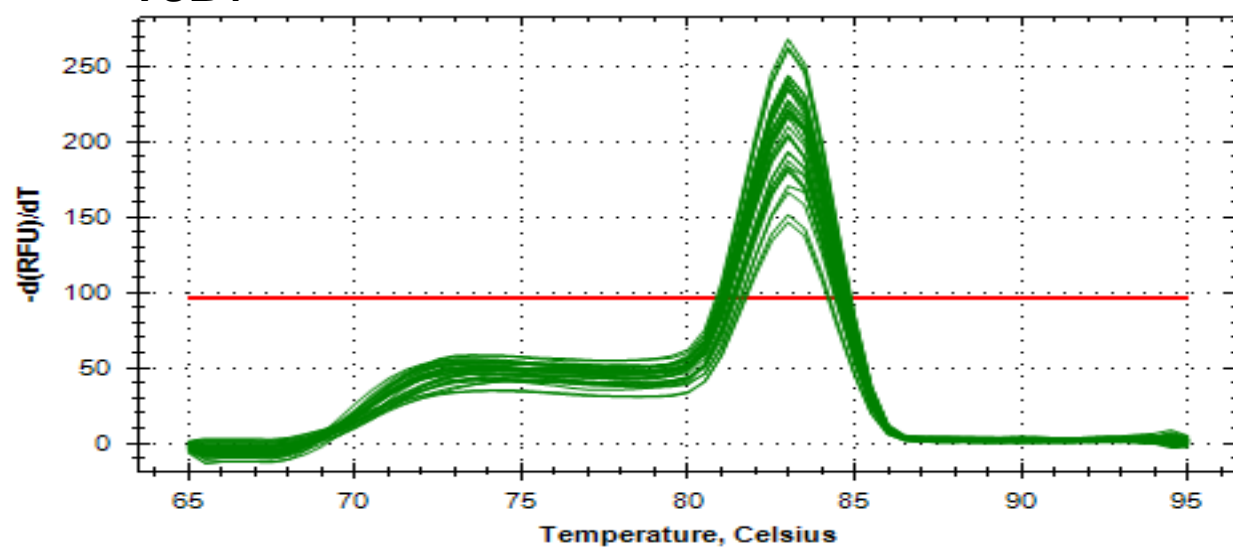

### ***RPL30***

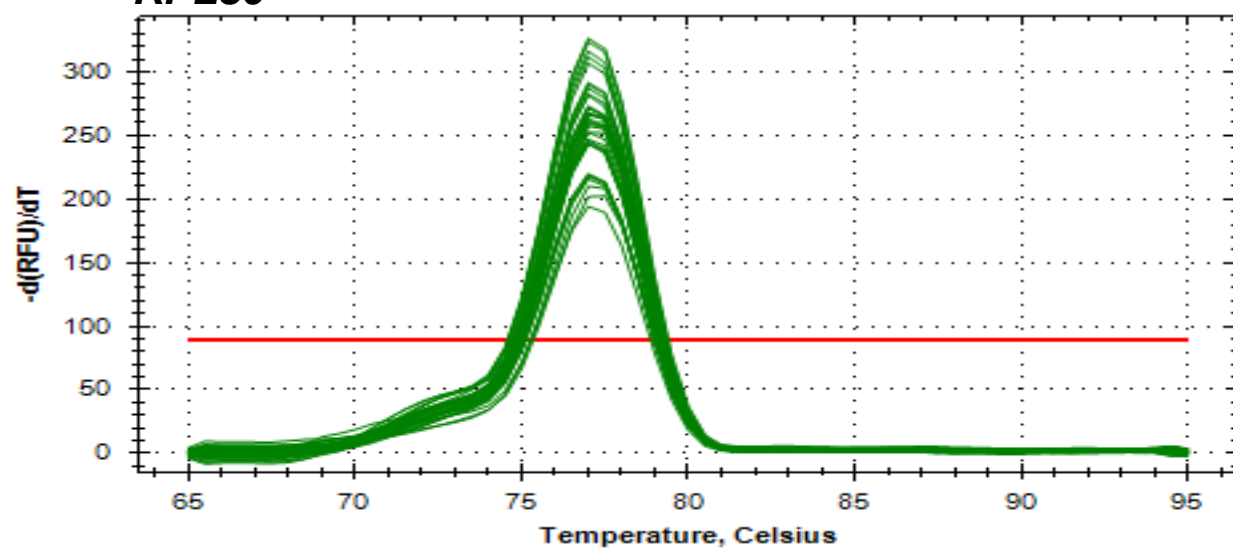

### ***TUA4***

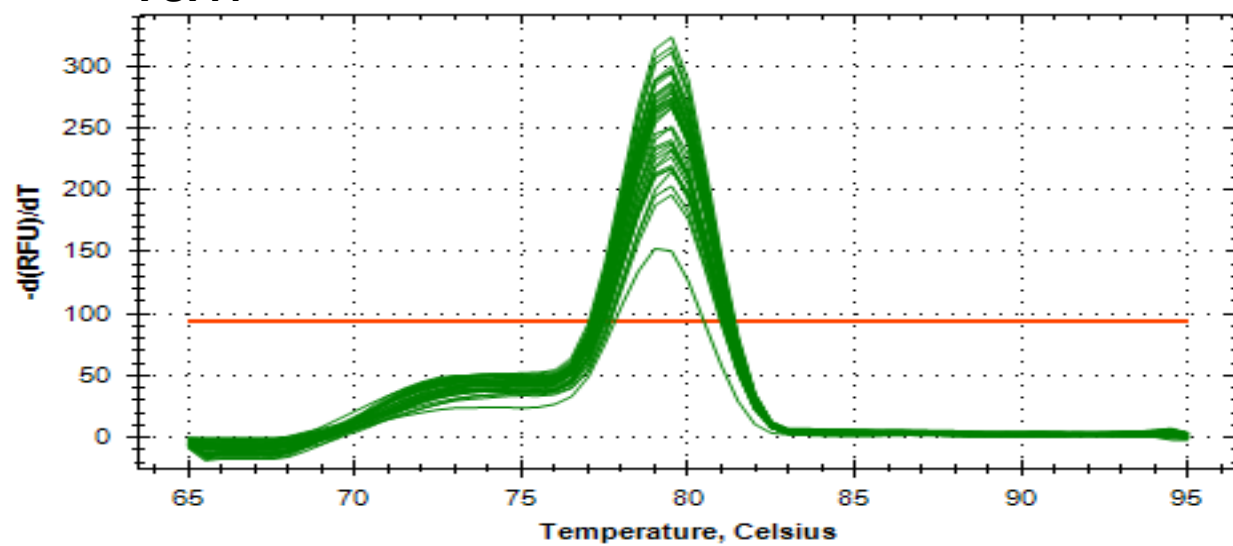

### ***GPDH***

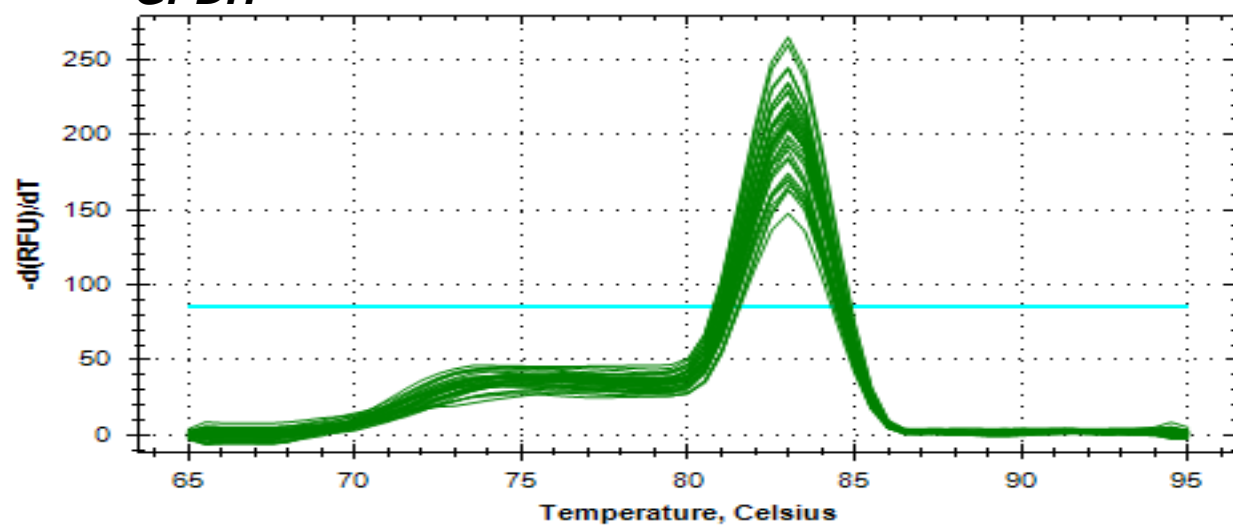

### ***TUA5***

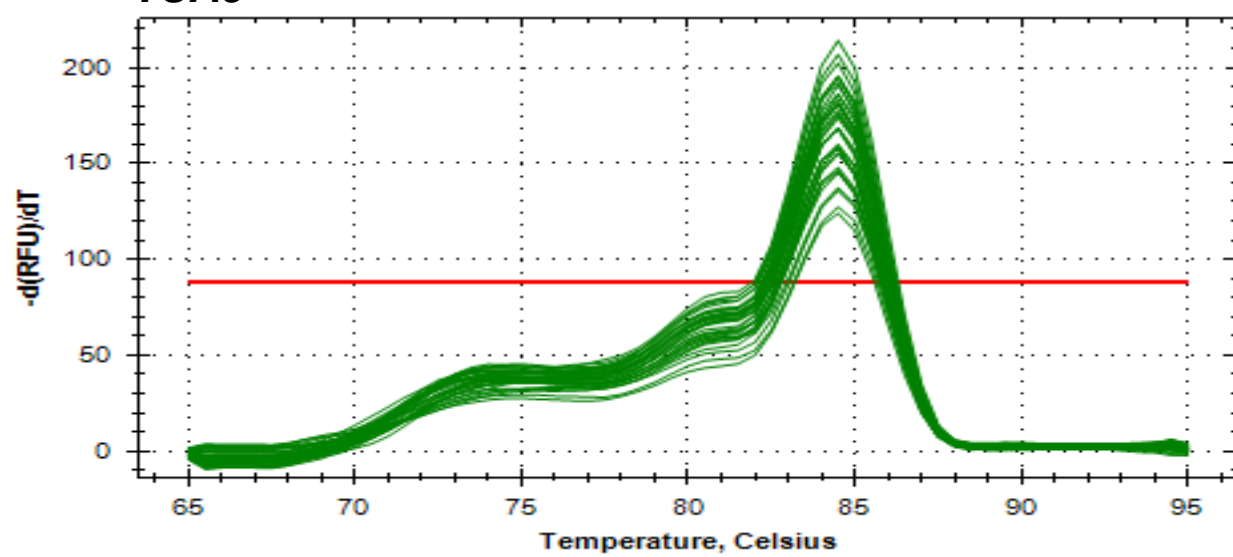

### ***ABCT***

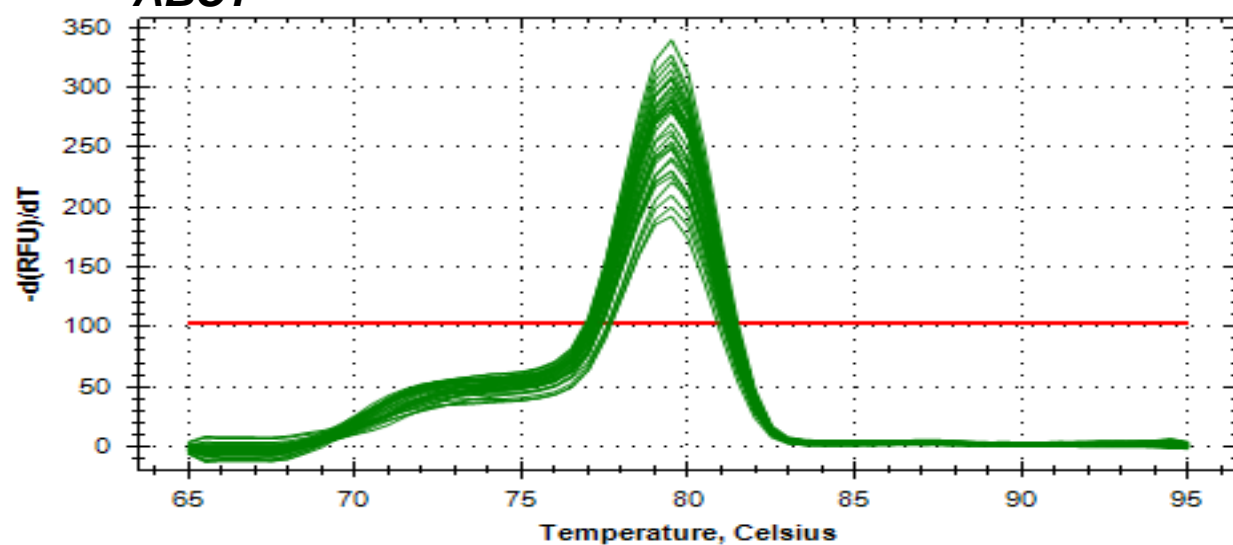

### ***EF1A***

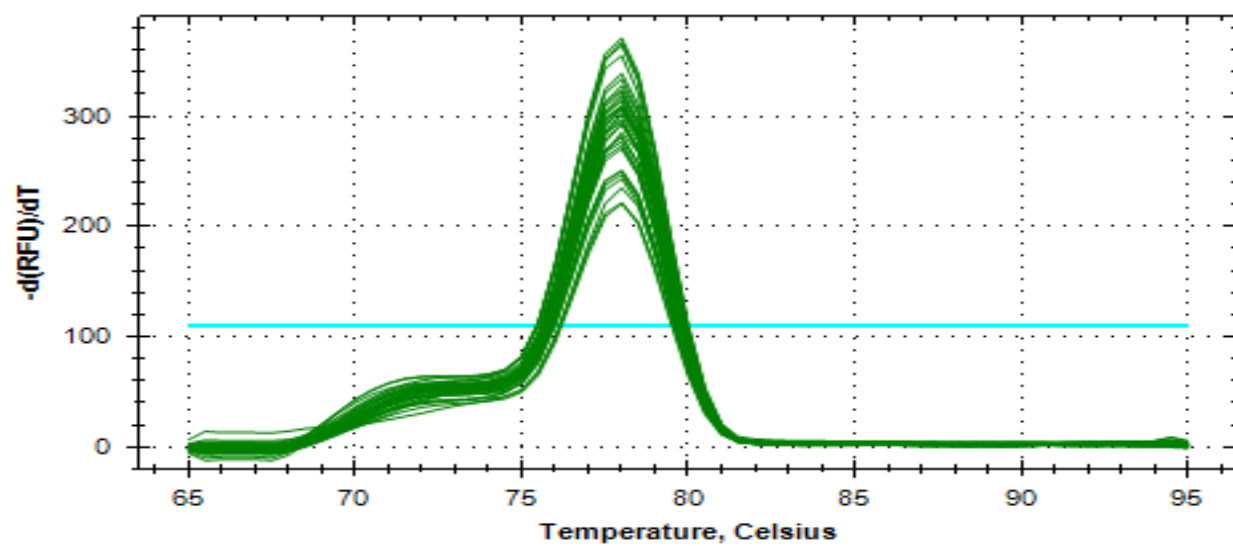

Supplement: S3 Fig — The melting curves are presented for primer pairs used to amplify tested reference genes in soybean under biotic stress. Details on tested reference genes and primer sequences are provided in Tables 1 and 2, respectively. (PDF) [file pone.0134890.s003.pdf]
